# Correction: Mechanistic details of the cobalt-mediated dehydrogenative dimerization of aminoquinoline-directed benzamides

**DOI:** 10.1039/d1sc90155a

**Published:** 2021-07-16

**Authors:** Li-Ping Xu, Elaine E. L.-N. Liu, John Bacsa, Manjaly J. Ajitha, Cora E. MacBeth, Djamaladdin G. Musaev

**Affiliations:** Cherry L. Emerson Center for Scientific Computation, Emory University Atlanta Georgia 30322 USA dmusaev@emory.edu; Department of Chemistry, Emory University Atlanta Georgia 30322 USA cmacbet@emory.edu; School of Chemistry and Chemical Engineering, Shandong University of Technology Zibo 255000 China

## Abstract

Correction for ‘Mechanistic details of the cobalt-mediated dehydrogenative dimerization of aminoquinoline-directed benzamides’ by Li-Ping Xu *et al.*, *Chem. Sci.*, 2020, **11**, 6085–6096, DOI: 10.1039/D0SC02066D.

The authors regret the omission of one of the authors, Manjaly J. Ajitha, from the original manuscript. The corrected list of authors and affiliations for this paper is as shown above.

The Royal Society of Chemistry apologises for these errors and any consequent inconvenience to authors and readers.

